# Diversity and functional analysis of gut microbiota reveal ecological adaptations in the inquilinism of *Ancistrotermes dimorphus* and its host *Macrotermes barneyi*

**DOI:** 10.3389/fmicb.2025.1587281

**Published:** 2025-06-25

**Authors:** Wenzhuo Lei, Zhifang Qin, Bao Jia, Wen Lu, Juan Yang, Qionghua Gao

**Affiliations:** ^1^Guangxi Key Laboratory of Agro-environmental and Agric-products Safety/National Demonstration Center for Experimental Plant Science Education, College of Agriculture, Guangxi University, Nanning, Guangxi, China; ^2^Termite Control of Nanning City, Nanning, Guangxi, China

**Keywords:** termite, cohabitation, gut microbiota, 16S rRNA sequencing, ecological adaptation

## Abstract

Inquilinism describes an interesting interspecific interaction in termite ecosystems wherein an inquiline species inhabits the host’s nest structure. In this context, gut microbiota play a crucial role in mediating the ecological relationship. The facultative inquiline *Ancistrotermes dimorphus* (Termitidae: Macrotermitinae) frequently inhabits nests of the host *Macrotermes barneyi* but can also establish independent colonies. We used 16S rRNA sequencing to compare the gut microbiota of *A. dimorphus* and *M. barneyi* in independent and inquilinism nests, assessing microbial diversity and composition. Gut microbiota diversity increased under inquilinism, with greater microbial similarity between *A. dimorphus* and *M. barneyi* in shared nests. Furthermore, inquilinism altered microbial function, increasing taxa linked to environmental adaptation while reducing those involved in energy metabolism, suggesting potential metabolic trade-offs. Beta diversity analysis indicated that inquilinism drives the gut microbiota adaptation between the host and inquiline. These findings reveal how gut microbiota mediates host-inquiline interactions, advancing our understanding of microbial adaptation in social insect symbiosis.

## 1 Introduction

Inquilinism is a notable phenomenon in termite ecosystems, where one species (inquiline) resides within the nest of another species (host) (Shellman-Reeve, 1997). This interaction is widespread among termites, with multiple inquiline species occasionally coexisting within a single host nest (Darlington, 2012). The relationship between host and inquiline can transit from facultative to obligate depending on the nest-building capabilities of the inquiline (Mathews, 1977). Facultative inquilines demonstrate the ability to establish independent nests or inhabit host colonies, whereas obligate inquilines (e.g., termites of the genera *Ahamitermes*, *Incolitermes*, *Macrotermes*, and *Termes*) lost their nest-building capacity (Cristaldo et al., 2014). These obligate inquilines typically occupy specialized chambers within host nests, avoiding both direct contact with hosts and encroachment into the royal chamber (Cristaldo et al., 2014).

The success of inquiline colonization is influenced by environmental factors and host colony dynamics (Rodrigues et al., 2021; Kechrid et al., 2025). Resource scarcity and large nest volumes facilitate invasion by weakening host defenses (Cristaldo et al., 2012; Marins et al., 2016), while host colony maturation into the reproductive phase reduces defensive vigilance (Cristaldo et al., 2012, 2016). During reproduction, host colonies prioritize resource allocation to alate production, resulting in fewer patrolling soldiers and creating temporal windows for inquiline infiltration (Rodrigues et al., 2021). Field studies demonstrate that such defense-reproduction trade-offs enable inquilines like *Inquilinitermes microcerus* to exploit host nests (*Constrictotermes cyphergaster*) with minimal resistance (Rodrigues et al., 2021).

Through inquilinism, inquilines reduce independent nest construction costs and gain access to shared resources and predation shelter (Florencio et al., 2013; Barbosa-Silva et al., 2016). However, prolonged coexistence drives ecological and behavioral adaptations in both host and inquiline species. Hosts experience altered caste ratios (e.g., soldier/worker ratio) and intensified resource competition (Rodrigues et al., 2018), while inquilines evolve strategies such as asexual reproduction under resource constraints (Timmermans et al., 2024) and chemical mimicry to evade host aggression (Cristaldo et al., 2014; Lorenzi et al., 2014; Hugo et al., 2020). Notably, inquilines can detect host defense signals to protect their own colonies (Cristaldo et al., 2014) and obtain food resources from host nests, including mound soil, nest walls, and even excreta (Florencio et al., 2013; Barbosa-Silva et al., 2016), which will further influence the composition of their gut microbiota.

Gut microbiota plays a critical role in host physiology and behavior, mediating core processes such as enabling digestion of diverse food sources, modulating host immune tolerance, and coordinating host behavior through cross-species social interactions (Macke et al., 2017). These microbial communities facilitate carbohydrate degradation and regulate physiological functions, aiding both host and inquiline in adapting to shared environments (Jannual et al., 2024). For instance, the facultative inquilines *Cavitermes tuberosus* exhibits significant overlap in gut microbiota composition with its host *Labiotermes labralis*, a phenomenon likely facilitated by horizontal microbial transfer during nest material consumption (Hellemans et al., 2019).

The facultative inquiline termite *Ancistrotermes dimorphus*, is distributed throughout Guangxi and Yunnan provinces of China, coexisting with host species including *Macrotermes barneyi*, *Macrotermes annandalei*, *Odontotermes formosanus* and *Odontotermes yunnanensis* (Wen et al., 2015). Research shows that *A. dimorphus* is more likely to nest in *M. barneyi* (82.76%) than in *O. formosanus* (36.78%) and other species (Lu et al., 2011). During inquilinism, *A. dimorphus* integrates its chambers and tunnels into the host’s nest structure (Lu et al., 2011). While previous studies have examined gut microbiota diversity in free-living and facultative inquiline termites, the specific influences of microbiota on inquilinism-related adaptations remains poorly understood.

In this study, we aim to investigate the gut microbiota of the inquiline *A. dimorphus* and its host *M. barneyi* under independent and inquilinism nesting using 16S rRNA sequencing. By analyzing microbial diversity and functional pathways, we aim to elucidate microbiota-driven adaptations that stabilize inquilinism, advancing our understanding of symbiotic evolution in social insects.

## 2 Materials and methods

### 2.1 Sample collection

**FIGURE 1:**
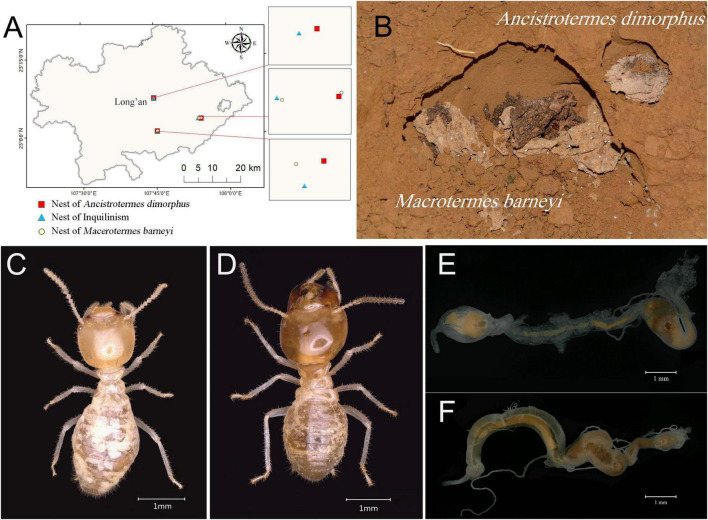
Sample information for the two termites. **(A)** Sampling locations in Guangxi Province. **(B)** Ecological photograph of the inquilinism nest, showing the chamber of the inquiline *A. dimorphus* and the host *M. barneyi*. **(C)** Worker of *A. dimorphus*. **(D)** Worker of *M. barneyi*. **(E)** Gut tissue of *A. dimorphus*. **(F)** Gut tissue of *M. barneyi*.

Termite samples were collected from several subtropical chestnut plantations (*Castanea mollissima*, canopy height 2-3 m) in Long’an County, Nanning, Guangxi, China (Figure 1A and Supplementary Table 1), a region characterized by a southern subtropical monsoon climate with an annual mean temperature of 21.8°C and precipitation of 1,301 mm. The lateritic red soil exhibited a pH 4.60 ± 0.62 and organic matter content of 2.68 ± 0.67% based on our surface soil survey. As *A. dimorphus* and *M. barneyi* construct subterranean nests with no obvious termite mound above the ground, nests were pre-mapped during April-June 2023 swarming seasons by identifying the species-specific flight holes. *A. dimorphus* constructs circular openings (4-8 mm diameter) of the flight hole flush with or slightly elevated above the ground surface, whereas *M. barneyi* had crescent-shaped flight holes (20-60 mm diameter) recessed 3-6 mm below ground. GPS-tagged nests were subsequently sampled from July to October in 2023, ensuring minimum 30 m spacing between collection points to avoid pseudoreplication.

Sampling included three independent nests of *A. dimorphus* (Adim), three independent nests of *M. barneyi* (Mbar), and three inquilinism nests containing the inquiline *A. dimorphus* (Adim-Inquiline) and the host *M. barneyi* (Mbar-Host). The two species were easily distinguished in shared nests by differences in fungus comb structure, soldier morphology, and worker morphology (Figure 1B). Termites of each species were collected along with the surrounding fungus comb and chamber using a shovel or by gently brushing individuals from chambers into the collection boxes. The studied *M. barneyi* nests represented mature colonies containing physogastric queen (mean body length: 2.23 ± 0.09 cm), which indicates an establishment of more than 4 years. In contrast, *A. dimorphus* colony size could not be determined due to their diffuse chamber networks and the absence of collected queens. Large workers with a minimum of 100 individuals per nest per species were collected for gut dissection. Upon collection, termite samples were morphologically verified, transferred into sterile centrifuge tubes, flash-frozen in liquid nitrogen, and stored at –80°C. Species identity was further confirmed through DNA barcoding of the mitochondrial COII gene, ensuring taxonomic accuracy.

### 2.2 Gut dissection

Large workers, which were stored for up to 3 months, were used for gut dissection. Workers were washed in PBS buffer to remove soil or other debris adhering to the surface of the specimen for 1 min and surface-sterilized with 75% ethanol for 30 s to 1 min. After a final rinse in PBS buffer, the termites were transferred into fresh PBS buffer and dissected under a stereomicroscope using sterile forceps to isolate the entire gut tissue (Figures 1C-F). For each sample, gut tissues from five workers were pooled into a 1.5 mL centrifuge tube for DNA extraction. Three biological replicates were prepared for each termite species and nesting strategy, resulting in a total of 36 pooled gut samples.

### 2.3 DNA extraction and sequencing of gut microbiota

Gut tissues were homogenized with 1 mL SLX-Mlus buffer and 500 mg magnetic beads, then shaken at 45 Hz for 250 s using a grinding mill. To avoid environmental DNA contamination, DNA extraction was conducted in an ultra-clean workbench. Total DNA was extracted using the E.Z.N.A.^®^ Soil DNA Kit (Omega Bio-Tek, United States) following the manufacturer’s protocol. DNA quality and concentration were evaluated by 1% agarose gel electrophoresis in 1 × TAE buffer at 100V for 20 min. The bacterial 16S rRNA V3-V4 region was amplified using the primers 341F (5′-CCTAYGGGRBGCASCAG-3′) and 806R (5′-GGACTACNNGGGTATCTAAT-3′). Each 20 μL PCR reaction contained 4 μL of 5 × FastPfu Buffer, 2 μL of 2.5 mM dNTPs, 0.8 μL of each Primer (5 μM), 0.4 μL of FastPfu Polymerase, 10 ng of template DNA, and sterile ddH_2_O. ddH_2_O served as a negative PCR control. The PCR amplification program was as follows: 95°C for 5 min; 28 cycles of 95°C for 30 s, 58°C for 30 s, and 72°C for 45 s; followed by a final extension at 72°C for 10 min. PCR products were confirmed via 2.0% agarose gel electrophoresis, purified, and used to construct sequencing libraries with the SMRTbell™ Template Prep kit 1.0 (Pacific Biosciences, United States). Sequencing was performed on the Illumina NovaSeq PE250 platform, generating a minimum of 55,000 paired-end reads per sample.

### 2.4 Data analysis

The raw sequencing data were subjected to quality control using Trimmomatic (v0.30) to remove low-quality reads and adapter sequences. Reads were quality-trimmed by removing bases from the 3′-end with quality scores below 20 using a sliding window approach (10-bp window size, truncation when average quality < 20). Reads shorter than 50 bp after trimming were discarded. Paired-end reads were merged into single sequences based on overlapping regions with a minimum overlap length of 10 bp. High-quality reads were assembled into contigs using FLASH (v1.2.7). Operational taxonomic units (OTUs) were clustered at by grouping sequences 97% sequence similarity using UPARSE (v7.1). Taxonomic classification of each sequence was performed using the RDP classifier (v2.2) with a confidence threshold of 80%, based on the Silva 16S rRNA database (v138). Microbial community composition was then analyzed at multiple taxonomic levels (phylum, class, order, family, genus, and species). To obtain species-level taxonomic information, representative OTU sequences were further analyzed using the UCLUST algorithm.

Rarefaction analysis using Mothur (v1.21.1) was conducted to assess sequencing depth sufficiency, followed by the calculation of alpha diversity indices, including the Chao1, ACE, Shannon, and Simpson diversity indices. Venn diagrams were drawn using the online tool “Draw Venn Diagram”^1^ to analyze overlapping and unique OTUs during the treatment processes. The beta diversity analysis was performed using Bray-Curtis distances for the principal coordinates analysis (PCoA) and non-metric multidimensional scaling (NMDS), one-way permutational analysis of variance (PERMANOVA) was performed using R vegan package to assess the statistically significant effects of termite groups on bacterial communities.

Linear discriminant analysis (LDA) and linear discriminant analysis effect size (LEfSe) were conducted to identify the biomarker. A Kruskal-Wallis sum-rank test was performed to examine the changes and dissimilarities among classes followed by LDA analysis to determine the size effect of each distinctively abundant taxa.

The phylogenetic Investigation of Communities by Reconstruction of Unobserved States (PICRUSt) program based on the Kyoto Encyclopedia of Genes and Genomes (KEGG) database was used to predict the functional alterations of microbiota in different samples. The OTU data obtained were used to generate BIOM files formatted as input for PICRUSt (v1.1.09) using the make.biom script usable in the Mothur. OTU abundances were mapped to Greengenes OTU IDs as input to speculate about the functional alteration of microbiota.

## 3 Results

### 3.1 Sequencing data and OTU analysis

A total of 36 gut samples were sequenced, with 34 samples retained after excluding 2 with significant discrepancies (with abnormal OTU distribution) in sequencing quality. The retained samples included 8 from independently nesting individuals of *A. dimorphus*, 9 from independently nesting individuals of *M. barneyi*, 9 from inquiline *A. dimorphus*, and 8 from host *M. barneyi*. A total of 2,070,670 clean reads were obtained, with an average of 60,902 clean reads per sample (minimum 55,206 clean reads).

**FIGURE 2:**
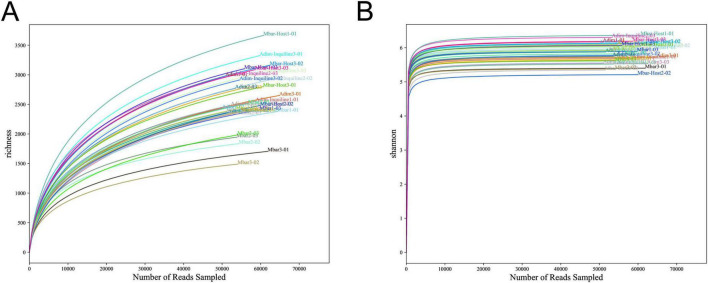
Rarefaction and Shannon-Wiener curves for gut microbial communities in four termite groups. **(A)** Rarefaction curves showing the sequencing depth and the accumulation of OTUs. **(B)** Shannon-Wiener index curves indicating the microbial diversity saturation.

Rarefaction curves (Figure 2A) demonstrated that the number of OTUs increased with sequencing depth, eventually reaching saturation, while the Shannon index rarefaction curves plateaued (Figure 2B). These results indicate that the sequencing depth was sufficient to capture the majority of gut microbiota diversity in these termite samples.

A total of 11,049 OTUs were annotated across all samples, representing 44 phyla, 103 classes, 219 orders, 310 families, and 559 genera (Supplementary Table 2). The OTU counts per group were: Adim (5,968), Mbar (5,172), Adim-Inquiline (6,494), and Mbar-Host (6,287), indicating a general increase in OTU richness in inquilinism-associated groups.

**FIGURE 3:**
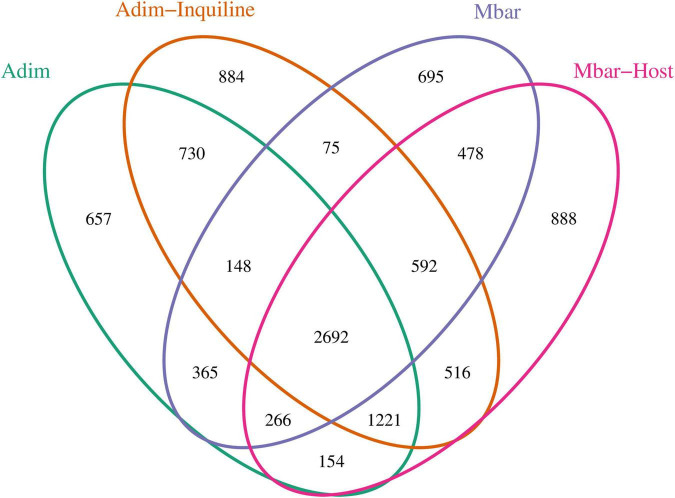
Venn diagram showing shared OTUs among four termite groups, indicating microbial adaptation in inquilinism colonies.

Analysis of shared OTUs analysis revealed 2,692 OTUs common across all four groups (Figure 3). Adim and Mbar shared 3,471 OTUs, while Adim-Inquiline and Mbar-Host shared 5,021 OTUs, highlighting increased microbial similarity between species in inquilinism nests. Similarly, Adim shared 4,791 OTUs with Adim-Inquiline, while Mbar shared 4,028 OTUs with Mbar-Host.

### 3.2 Alpha diversity

**FIGURE 4:**
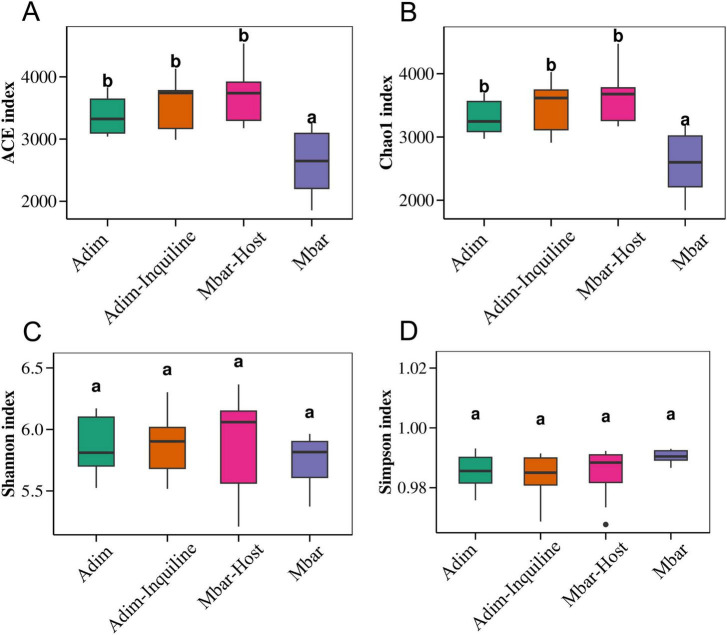
Boxplots of alpha diversity indices for gut microbial communities in four termite groups. **(A)** ACE index; **(B)** Chao1 index; **(C)** Shannon index; **(D)** Simpson index.

Alpha diversity index revealed significant differences among groups (Supplementary Table 3). The ACE and Chao1 indices were significantly higher in Adim, Adim-Inquiline and Mbar-Host compared to Mbar (*p* < 0.05, Tukey’s HSD test; Figures 4A,B), suggesting greater microbial richness in *A. dimorphus* and the host under inquilinism nests. In contrast, Shannon (Adim-Inquiline vs. Adim: *p* = 0.99, Mbar vs. Adim: *p* = 0.82, Mbar-Host vs. Adim: *p* = 0.99, Mbar vs. Adim-Inquiline: *p* = 0.76, Mbar-Host vs. Adim-Inquiline: *p* = 0.99, Mbar-Host vs. Mbar: *p* = 0.77; Tukey’s HSD test, Figure 4C) and Simpson (Adim-Inquiline vs. Adim: *p* = 0.95, Mbar vs. Adim: *p* = 0.45, Mbar-Host vs. Adim: *p* = 0.99, Mbar vs. Adim-Inquiline: *p* = 0.18, Mbar-Host vs. Adim-Inquiline: *p* = 0.99, Mbar-Host vs. Mbar: *p* = 0.33; Tukey’s HSD test, Figure 4D) indices showed no significant differences across groups (Figures 4C,D), indicating similar microbial community evenness.

### 3.3 Gut microbial diversity

**FIGURE 5:**
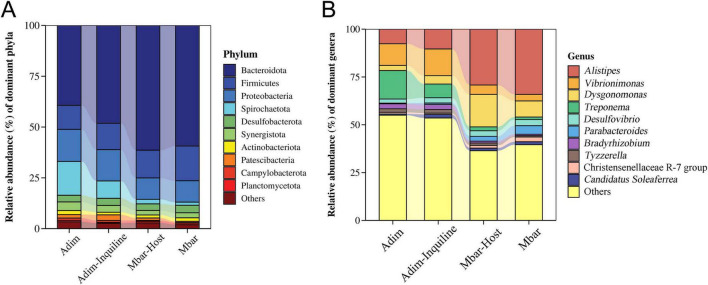
Relative abundance of gut microbial communities at the **(A)** phylum and **(B)** genus level in four termite groups.

At the phylum level, the gut microbiota of all groups was dominated by Bacteroidota, Proteobacteria, and Firmicutes, with Bacteroidota showing the highest abundance (Figure 5A). In *M. barneyi*, Bacteroidota accounted for 59.31% (Mbar) and 61.37% (Mbar-Host), significantly higher than in *A. dimorphus* (Adim 39.32%, Adim-Inquiline 48.09%, *p* < 0.05, Kruskal-Wallis test). Proteobacteria was more abundant in *A. dimorphus* (Adim 15.78%, Adim-Inquiline 15.41%) than in *M. barneyi* (Mbar 10.54%, Mbar-Host 10.51%, *p* < 0.05, Kruskal-Wallis test). Firmicutes, however, showed higher abundance in *M. barneyi* (Mbar 17.11%, Mbar-Host 13.69%) compared to *A. dimorphus* (Adim 11.82%, Adim-Inquiline 13.03%, *p* < 0.05, Kruskal-Wallis test). Both Proteobacteria and Firmicutes play essential roles in nutrient absorption within the termite guts and are closely related to host physiological activities, including reproduction, growth, and nitrogen fixation. In contrast, Spirochaetota, critical for lignocellulose degradation, was significantly more abundant in *A. dimorphus* (Adim 16.65%, Adim-Inquiline 8.51%) than in *M. barneyi* (Mbar 1.53%, Mbar-Host 2.24%, *p* < 0.05, Kruskal-Wallis test), highlighting species-specific ecological roles of gut microbiota (Supplementary Table 4).

At the genus level, *Alistipes*, *Vibrionimonas*, and *Dysgonomonas* were predominant across groups (Figure 5B and Supplementary Table 5). *Alistipes* was most abundant in *M. barneyi* (Mbar 34.10%, Mbar-Host 29.22% vs. Adim 7.63%, Adim-Inquiline 10.35%, *p* < 0.05, Kruskal-Wallis test), while *Vibrionimonas* was more abundant in *A. dimorphus* (Adim 11.30%, Adim-Inquiline 13.98% vs. Mbar 3.41%, Mbar-Host 4.90%, *p* < 0.05, Kruskal-Wallis test). The abundance of *Dysgonomonas* was significantly higher in Mbar-Host than in Mbar (Mbar 8.41% vs. Mbar-Host 16.97%, *p* < 0.05, Kruskal-Wallis test), suggesting increased lignocellulose consumption by *M. barneyi* after the invasion of *A. dimorphus*. In addition, *Treponema*, a genus within Spirochaetota (which is richer in *A. dimorphus*), was significantly reduced in Adim-Inquiline (Adim 14.88%, Adim-Inquiline 7.19%) compared to *M. barneyi* (Mbar 1.32%, Mbar-Host 2.05%, *p* < 0.05, Kruskal-Wallis test). This reduction in Adim-Inquiline aligns with the overall decrease in Spirochaetota, likely reflecting changes in microbial community dynamics due to inquilinism (Supplementary Table 4).

### 3.4 Beta diversity

**FIGURE 6:**
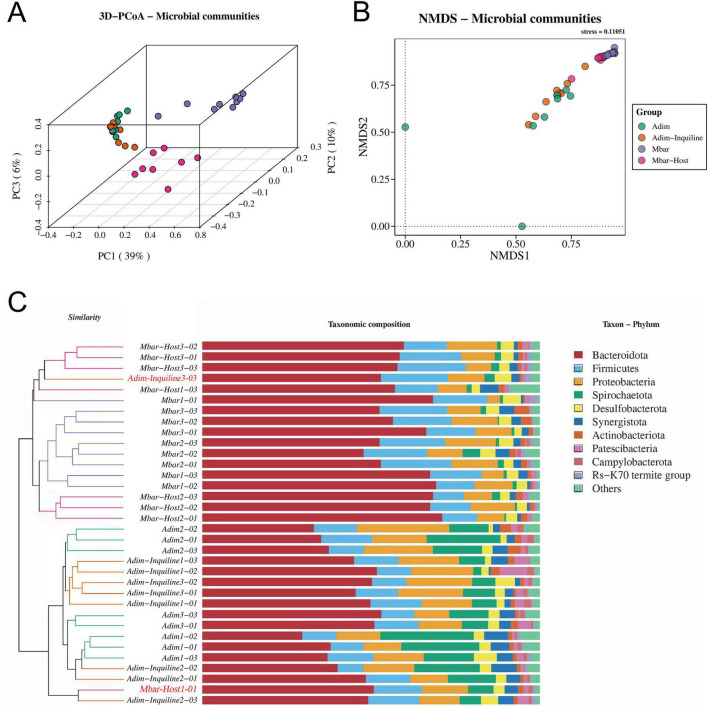
Beta diversity of gut microbiota across four termite groups (Based on Bray-Curtis distance). **(A)** 3D PCoA analysis of gut microbiota community similarity among four termite groups. The Adim and Adim-Inquiline groups exhibit higher intra- and inter-group similarity in bacterial communities, whereas Mbar and Mbar-Host show relatively weaker intra-group similarity. **(B)** NMDS analysis (Stress = 0.11051, indicating good reliability of the analysis). The Mbar and Mbar-Host groups are tightly clustered, while Adim and Adim-Inquiline groups exhibit a wider dispersion. **(C)** UPGMA clustering of the top 10 most abundant microbial phyla. Branches with the same color represent samples from the same termite group. Notably, Adim-Inquiline3-03 and Mbar-Host1-01 (marked in red) show higher similarity to Mbar-Host and Adim-Inquiline, respectively.

Beta diversity analysis revealed distinct differences in microbial community composition across groups. The 3D-PCoA plot (Figure 6A) showed that PC1, PC2, and PC3 explained 39, 10, and 6% of the variance, respectively (Adonis test, *R^2^* = 0.4629, *p* = 0.001). Samples from *A. dimorphus* and *M. barneyi* were distinct from one another, indicating significant interspecies differences in gut microbial composition. Within each species, different nesting strategies also contributed to variation, with samples from independent nesting and inquilinism forming separate clusters. Interestingly, the samples from the inquilinism nests (Adim-Inquiline and Mbar-Host) tended to close to each other, suggesting an adaptation in their microbial communities, potentially driven by shared nest environments and microbial transmission.

NMDS analysis (Figure 6B) confirmed the PCoA results, showing close proximity between Adim-Inquiline and Mbar-Host samples, which suggested adaptation in their gut microbiota due to shared environmental conditions and microbial transmission within the inquilinism nest. By contrast, Adim and Mbar samples were more widely distributed, reflecting greater intergroup variability.

UPGMA clustering (Figure 6C) further supported these findings, with Adim-Inquiline and Mbar-Host samples clustering together, indicating microbial similarity within inquilinism nests. Samples from independently nesting Adim and Mbar formed distinct branches, highlighting the role of inquilinism in driving microbial adaptation.

### 3.5 Differential analysis

**FIGURE 7:**
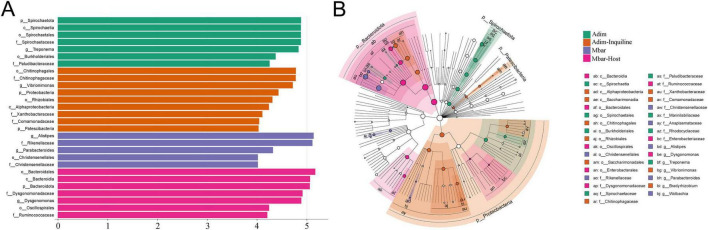
Differential analysis of gut microbiota in four termite groups. **(A)** Linear discriminant analysis (LDA) results (*P* < 0.05) showing species with significant abundance differences across four termite groups. The length of the bars represents the impact size of the differential species (LDA score). **(B)** LEfSe evolutionary branching diagram from phylum to genus level. The diagram highlights key microbial species playing prominent roles in the Mbar-Host and Adim-Inquiline groups.

Linear discriminant analysis (LDA) identified key microbial taxa contributing to differences among groups (Figure 7A). In Adim, Spirochaetota, Burkholderiales, and Paludibacteraceae were the most influential taxa (LDA > 4). In Adim-Inquiline, *Chitinophaga*, *Vibrionimonas*, Proteobacteria, and Rhizobiales showed significant contributions (LDA > 4). The relative abundance of microorganisms indicates that the core microbiota in Adim are primarily responsible for enhancing the host’s capacity to exploit the lignocellulose-rich diets and stabilizing the nest microenvironment, while Adim-Inquiline groups evolved to metabolize host-modified substrates and developed stress tolerance mechanisms, reducing direct competition with hosts. In Mbar, *Alistipes* and Rikenellaceae were the most influential taxa (LDA > 5), while *Parabacteroides* and Christensenellaceae also played notable roles. In Mbar-Host, *Bacteroides*, *Dysgonomonas*, *Oscillospira*, and Ruminococcus exhibited higher relative abundances, reflecting their roles in lignocellulose degradation and short-chain fatty acid production. These functions are critical for energy acquisition and host gut health. The LEfSe evolutionary branching diagram (Figure 7B) highlighted microbial shifts across groups, particularly the increased abundance of lignocellulose-degrading taxa in Mbar-Host and Adim-Inquiline, emphasizing ecological adaptation to inquilinism.

### 3.6 Functional predictions

**FIGURE 8:**
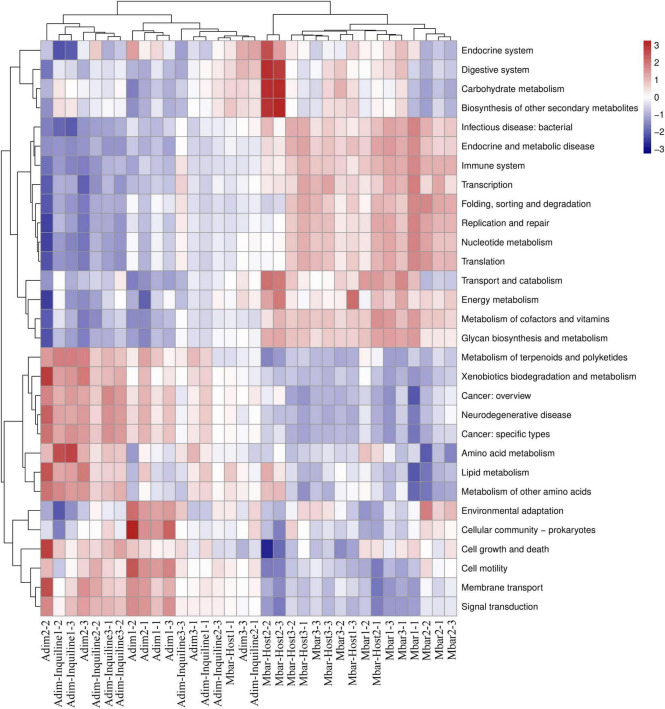
Functional prediction heatmap for gut microbiota in four termite groups (level 2). The clustering of functional differences indicates changes in the microbial functions during the transition from independent nesting to inquilinism. Specific functions (upregulated in red, downregulated in blue) reflect ecological adaptation of the gut microbiota to the shift in nesting strategy.

The 16S rRNA sequencing data were analyzed by PICRUSt to predict the functions of termites from different nesting strategies. Functional predictions revealed significant shifts in microbial functions associated with inquilinism (Mann-Whitney U test, *p* < 0.05; Figure 8). In Adim-Inquiline, microbes related to digestive systems, carbohydrate metabolism, and biosynthesis of other secondary metabolites were more abundant compared to those in Adim. Additionally, there was a marked increase in the abundance of microbes involved in amino acid metabolism, xenobiotics biodegradation and metabolism, and lipid metabolism. In contrast, microbial functions related to signal transduction, membrane transport, cell motility, endocrine system, and environmental adaptation were decreased.

In Mbar-Host, microbes involved in metabolism of cofactors and vitamins, glycan biosynthesis and metabolism, transport and catabolism, endocrine systems, digestive systems, carbohydrate metabolism, biosynthesis of secondary metabolites, and energy metabolism were more abundant compared to those in Mbar, reflecting the increased metabolic demands imposed by inquilinism. Meanwhile, microbes associated with immune systems, transcription and translation, nucleotide metabolism, cell growth and death, environmental adaptation, membrane transport, and signal transduction were decreased (Figure 8).

These results suggest that inquilinism drives microbial functional shifts, enabling *A. dimorphus* to adapt to shared nest environments while imposing additional biosynthetic and metabolic burdens on *M. barneyi*. This highlights the intricate ecological interplay between host and inquiline species.

## 4 Discussion

The gut microbiota of termites constitute an indispensable component and are perpetuated through dual transmission pathways: vertical (parent-to-offspring) and horizontal (interspecific or environmental acquisition) (Bourguignon et al., 2018; Arora et al., 2022). Vertical transmission serves as the primary mechanism, ensuring the intergenerational retention of core microbial lineages via trophallaxis or coprophagy (Rahman et al., 2015). Horizontal transmission, facilitated through heterospecific cannibalism or environmental exposure, is corroborated by genomic evidence of widespread interspecies bacterial transfer across evolutionary timescales (Bourguignon et al., 2018). This dual transmission strategy ensures both functional continuity and ecological flexibility, allowing termites to thrive in diverse niches.

Nest environments were found to significantly influence the composition and functional dynamics of insect gut microbiota (Liberti et al., 2024). Our results demonstrate that the number of shared OTUs between *A. dimorphus* inquilines and *M. barneyi* hosts substantially exceeded those in independent nest groups (Figure 3), suggesting that horizontal transmission and environmental selection drive microbial community restructuring in shared nests. This aligns with observations in facultative inquiline *C. tuberosus*, which exhibited the highest number of OTUs with its primary host *Labiotermes labralis* (Hellemans et al., 2019). The shared nest environment imposes similar ecological pressures on both species, driving the adaptation of their gut microbiota. While comparative studies on termite host-inquiline gut microbiomes remain limited, analogous patterns are evident across taxa. For instance, Opiliones harvestmen exhibit gut microbiota-mediated adaptation to resource-limited environments (Zhu et al., 2024), and the symbiotic lifestyle of *Myrmecophilous* ants directly shapes the gut microbiome of cohabiting *Neoasterolepisma* silverfish (Parmentier et al., 2024). These parallels underscore the critical role of environmental factors and microbial exchange in facilitating cohabitation adaptation. By utilizing shared resources and environmental microbes, both host and inquiline species enhance their metabolic versatility, thereby improving resilience to environmental fluctuations.

The interaction between host and inquiline microbiota is a key factor in shaping the gut microbiomes of both species and their ecological adaptation (Wang et al., 2023). Functional predictions revealed that *A. dimorphus* in the inquilinism environment downregulated microbiota taxa associated with core energy metabolism pathways but upregulated taxa linked to carbohydrate and amino acid metabolism, as well as the biosynthesis of other secondary metabolites. The gut microbiota of *A. dimorphus* inquiline actively participates in the degradation of lignocellulose and other recalcitrant compounds (Barcoto et al., 2020; Xie et al., 2024). This shift reflects metabolic adjustments that reduce energy expenditure while optimizing resource utilization. Conversely, the host *M. barneyi* may exhibit enhanced functions in carbohydrate metabolism and polysaccharides biosynthesis, but could experience a trade-off characterized by potentially reduced microbial functions associated with transcription, translation, cell growth, and immune system activity. Eusocial insects can employ various behavioral and physiological defense mechanisms to avoid, resist, and tolerate pathogen infections within their closely related and densely populated communities. However, this process relies heavily on carbohydrate metabolism to generate the energy required (Hassan et al., 2022; Liu et al., 2023). These findings may indicate that the host termite could potentially incur heightened metabolic demands to sustain inquiline species, while also possibly experiencing reduced immune capacity as a compensatory mechanism (Sumner et al., 2003), reflecting a potential trade-off in resource allocation strategies.

The role of lignocellulose degradation in termite gut microbiota may suggest potential functional interactions between host and inquiline species. *Treponema* is critical for xylan degradation and acetate production providing energy for the host (Kakumanu et al., 2018; Chouaia et al., 2019). A mutualistic relationship exists between *Treponema* and the host termites, characterized by its physiological capabilities, including CO*2* reducing acetogenesis. The resulting acetate fulfills a significant portion of the host’s respiratory energy requirements (Tokuda, 2021). In this study, *Treponema* reduction in *A. dimorphus* inquiline may indicate a shift in carbohydrate fermentation strategies, but further metabolic analysis is needed to confirm changes in respiratory energy reliance. The observed increase in *Dysgonomonas* and *Ruminococcus* abundance in *M. barneyi* hosts could imply possible heightened demands on host-mediated xylan and plant material degradation. Bacteria from *Dysgonomonas* are facultative heterotrophs capable of utilizing lignocellulose-derived polysaccharides for growth. They possess a substantial repertoire of genes involved in glycosylation processes, making them well-suited to thrive in the lignocellulose-rich hindgut of termites (Bridges and Gage, 2021). Such functional shifts might reflect inquiline species’ conditional dependence on host metabolic processes for nutrient acquisition, potentially indicating microbial metabolism’s ecological role in termite adaptation to inquilinism environments (Su et al., 2015; Arab et al., 2024).

Overall, this study demonstrates that gut microbiota facilitates host-inquiline interactions and ecological adaptation in termite inquilinism, fundamentally advancing our understanding of symbiotic plasticity in social insects. While prior comparative studies mostly focused on free-living termite species, we provide a detailed analysis of gut microbial remodeling in a facultative inquilinism system, contrasting the host *M. barneyi* and inquiline *A. dimorphus*. Future investigations in microbial transmission mechanisms and functional reciprocity will further elucidate how symbiont networks stabilize termite inquilinism through niche complementarity.

## Data Availability

The datasets presented in this study can be found in online repositories. The names of the repository/repositories and accession number(s) can be found at: https://www.ncbi.nlm.nih.gov/, PRJNA1221240.

## Footnotes

1
http://bioinformatics.psb.ugent.be/webtools/Venn
