# Supplementary material for: Diversity and functional analysis of gut microbiota reveal ecological adaptations in the inquilinism of Ancistrotermes dimorphus and its host Macrotermes barneyi
Source: Front Microbiol. 2025 Jun 25;16:1587281. doi: 10.3389/fmicb.2025.1587281 (PMC12239989; doi:10.3389/fmicb.2025.1587281)
Supplement: Supplementary file 1 [file Table_1.docx]

***Supplementary Material of***

Diversity and functional analysis of gut microbiota reveal ecological adaptations in the inquilinism of *Ancistrotermes dimorphus* and its host *Macrotermes barneyi*

**Supplementary Table 1** Nest GPS locations

| **Type** | **Collection time** | **Longitude** | **Latitude** |
| --- | --- | --- | --- |
| **Mbar** | 2023.7.27 | 107°45'19.964"E | 23°7'19.195"N |
|  | 2023.8.3 | 107°44'49.177"E | 23°2'29.613"N |
|  | 2023.10.13 | 107°44'53.575"E | 23°2'30.331"N |
| **Adim** | 2023.10.13 | 107°44'49.694"E | 23°3'23.964"N |
|  | 2023.10.13 | 107°44'45.038"E | 23°3'20.493"N |
|  | 2023.8.3 | 107°44'51.505"E | 23°2'26.620"N |
| **Inquilinism** | 2023.10.13 | 107°45'13.949"E | 23°7'15.965"N |
|  | 2023.8.3 | 107°47'27.509"E | 23°8'2.507"N |
|  | 2023.8.3 | 107°45'19.058"E | 23°7'13.213"N |

**Supplementary Tabl**e 2 The statistics of gut microbiota in four groups of termites at various taxonomic levels

|  | **Phylum** | **Class** | **Order** | **Family** | **Genus** |
| --- | --- | --- | --- | --- | --- |
| **Adim** | 33 | 65 | 147 | 204 | 305 |
| **Adim-Inquiline** | 28 | 58 | 121 | 164 | 238 |
| **Mbar-Host** | 33 | 64 | 138 | 185 | 266 |
| **Mbar** | 28 | 66 | 138 | 196 | 277 |

**Supplementary Table 3** Statistical analysis of alpha diversity indices

|  | **Observed species** | **Chao1** | **ACE** | **Shannon** | **Simpson** |
| --- | --- | --- | --- | --- | --- |
| **Adim** | 2593 | 3302.077 | 3376.599 | 5.863 | 0.985 |
| **Adim-Inquiline** | 2766 | 3471.076 | 3543.824 | 5.875 | 0.984 |
| **Mbar-Host** | 2870 | 3633.706 | 3700.275 | 5.875 | 0.985 |
| **Mbar** | 2070 | 2598.248 | 2625.773 | 5.736 | 0.990 |

**Supplementary Table 4** The top ten microbial phyla by relative abundance in the four groups of termites (Kruskal-Wallis test, *p* < 0.05)

| **Sample**  **ID** | **Bacteroidota** | **Firmicutes** | **Proteobacteria** | **Spirochaetota** | **Desulfobacterota** | **Synergistota** | **Actinobacteriota** | **Pastescibacteria** | **Campylobacterota** | **Planctomycetota** |
| --- | --- | --- | --- | --- | --- | --- | --- | --- | --- | --- |
| **Adim** | 39.32 a | 11.82 a | 15.78 a | 16.65 a | 3.26 a | 4.25 a | 1.98 a | 1.72 ab | 1.15 a | 0.69 a |
| **Adim-Inquiline** | 48.09 a | 13.03 ab | 15.41a | 8.51b | 3.53 a | 3.33 a | 1.30 a | 2.77 a | 0.94 ab | 0.44 a |
| **Mbar-Host** | 61.37 b | 13.69 ab | 10.51a | 2.24 c | 3.20 a | 2.26 a | 1.50 a | 1.22 ab | 0.39 b | 0.80 a |
| **Mbar** | 59.31 b | 17.11 b | 10.54a | 1.53 c | 3.71 a | 2.48 a | 1.75 a | 0.58 b | 0.30 b | 0.76 a |

**Supplementary Table 5** The top ten microbial genera by relative abundance in the four groups of termites (Kruskal-Wallis test, *p* < 0.05)

| **Sample**  **ID** | **Alistipes** | **Vibrionimonas** | **Dysgonomonas** | **Treponema** | **Desulfovibrio** | **Bradyrhizobium** | **Parabacteroides** | **Tyzzerella** | **Christensenellaceae R-7 group** | **Candidatus Soleaferrea** |
| --- | --- | --- | --- | --- | --- | --- | --- | --- | --- | --- |
| **Adim** | 7.63 a | 11.30 a | 2.72 a | 14.88 a | 2.15 a | 2.61 a | 0.36 a | 1.86 a | 0.86 a | 0.63 a |
| **Adim-Inquiline** | 10.35 a | 13.98 a | 4.36 a | 7.19 b | 2.72 a | 2.70 a | 0.70 a | 1.87 a | 0.69 a | 1.86 a |
| **Mbar-Host** | 29.22 b | 4.90 b | 16.97 b | 2.05 bc | 2.92 a | 0.96 b | 2.51 c | 1.25 ab | 1.42 a | 1.37 a |
| **Mbar** | 34.10 b | 3.41 b | 8.41 a | 1.32 c | 3.21 a | 0.74 b | 4.47 b | 0.71 b | 2.48 b | 1.53 a |
